# Implementierung eines individualisierbaren tabletbasierten Trainingsprogramms im Anschluss an eine Parkinson-Komplexbehandlung in der Häuslichkeit – Erfolgsfaktoren und Barrieren

**DOI:** 10.1007/s00115-021-01202-0

**Published:** 2021-10-07

**Authors:** Lynn Wagner, Ruth Deck

**Affiliations:** Institut für Sozialmedizin und Epidemiologie, Universität zu Lübeck, Ratzeburger Allee 160, 23562 Lübeck, Deutschland

**Keywords:** Idiopathisches Parkinson-Syndrom, Mixed-Methods-Studie, Nachsorge, Körperliche Aktivität, Videogestützes Training, Idiopathic Parkinson’s disease, Mixed methods study, Aftercare, Physical activity, Video-based Training

## Abstract

**Hintergrund:**

Regelmäßige körperliche Aktivität ist bei Morbus Parkinson von großer Relevanz. Im Rahmen des Projekts „Individualisiertes Trainingsprogramm für Parkinson-Patienten“ (ParkProTrain) wurde ein individualisierbares tabletbasiertes Programm zur Unterstützung eines individuellen Eigentrainings entwickelt und über einen Zeitraum von 9 Monaten in der Häuslichkeit erprobt. Patienten wurden zur Machbarkeit der Nutzung und zur Zufriedenheit befragt.

**Methodik:**

Patienten, die das Programm im Anschluss an eine stationäre multimodale Komplexbehandlung bei Morbus Parkinson (Parkinson-Komplexbehandlung, PKB) in der Häuslichkeit nutzten, wurden zu zwei Zeitpunkten (9 und 36 Wochen nach PKB) im Rahmen qualitativer Interviews befragt.

**Ergebnisse:**

Das Programm half den Patienten über die gesamte Interventionszeit hinweg, motiviert zu bleiben. Es bot ihnen sowohl Struktur als auch Flexibilität für die Entwicklung einer eigenen Trainingsroutine. Als positive Aspekte (Erfolgsfaktoren) wurden u. a. benannt: die Einführungsseminare in der Klinik, die enge Betreuung während der Interventionszeit, die Fundiertheit und der Parkinson-Bezug, die Machbarkeit und die Handhabbarkeit des Programms sowie die individualisierten Trainingspläne. Patienten geben aber auch Hinweise, wie das Programm noch optimiert werden könnte (Barrieren).

**Diskussion:**

Das Programm unterstützt die Patienten bereits erfolgreich mit einer Vielzahl aktivitätsfördernder Ideen. Es konnten weitere Empfehlungen für zukünftige Vorhaben abgeleitet werden, die verstärkt beachtet werden sollten: Parkinson-Spezifität und Individualisierbarkeit des Programms, örtlich und zeitlich flexibles Training, enge und persönliche Betreuung über die gesamte Studienlaufzeit hinweg und eine einfach erlernbare und handhabbare Technik.

## Abstract (en)

**Background:**

Regular physical activity is of great relevance in Parkinson’s disease. As part of the project “Individualized training program for Parkinson’s disease patients” (ParkProTrain), a tablet-based program was developed to support individual self-training. It has been tested over a period of 9 months in domestic settings. Patients were asked about the feasibility of use and satisfaction.

**Methods:**

Patients who used the program at home following inpatient Parkinson’s complex treatment (PCT) were interviewed twice (9 and 36 weeks after PCT).

**Results:**

The program helped patients to stay motivated throughout the interventional period. It provided them with both structure and flexibility to develop their own training routine. The following were named as positive aspects (success factors), including: the introductory seminars in the clinic, the close supervision during the interventional period, the soundness and Parkinson’s specificity, the feasibility and manageability of the program and the individualized training plans; however, patients also provided information on how the program could still be optimized (barriers).

**Discussion:**

The program has already successfully implemented a number of aspects that promote activity. Further recommendations were derived for future use, to which more attention should be paid: Parkinson’s specificity and individualization of the program, flexible training in terms of location and time, close and personal support throughout the study period and the technique should be designed to be easy to learn and manage.

Eine Vielzahl an Studien belegt: Körperliche Aktivität bei Morbus Parkinson ist ein wichtiger Therapiebaustein [2]. Dennoch sind viele Betroffene in ihrem Alltag überwiegend inaktiv [20]. Es bedarf geeigneter Konzepte, die Patienten mit Parkinson (PmP) unterstützen, ein regelmäßiges individualisiertes Eigentraining zu etablieren. Im Rahmen der ParkProTrain-Studie wurde zu diesem Zweck ein individualisierbares tabletbasiertes Trainingsprogramm entwickelt [16] und eingesetzt. Mittels qualitativer Interviews lassen sich wesentliche Erfolgsfaktoren und Barrieren der Umsetzung im Alltag identifizieren. Die Finanzierung des Projekts erfolgte aus Mitteln des Innovationsfonds des Gemeinsamen Bundesauschusses (G-BA).

## Hintergrund und Fragestellung

Parkinson bedeutet für die Erkrankten ein großes körperliches und psychisches Leid. Die Krankheit verursacht hohe Kosten für das deutsche Gesundheitssystem [14]. Zahlreiche Studien belegen positive Effekte von Physiotherapie auf motorische und nichtmotorische Symptome der Erkrankung [3, 6]. Sie gewinnt bei PmP zunehmend an Bedeutung [2]. Eine regelmäßig an die individuelle Situation des Betroffenen angepasste körperliche Aktivität wird beim idiopathischen Parkinson-Syndrom (IPS) auch in der entsprechenden S3-Leitlinie empfohlen (Deutsche Gesellschaft für Neurologie, 2016). Dennoch sind die meisten PmP kaum körperlich aktiv [20].

**Figure 1:**
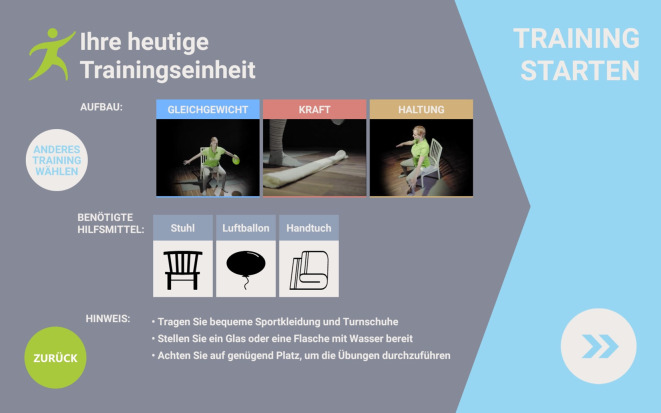

**Figure 2:**
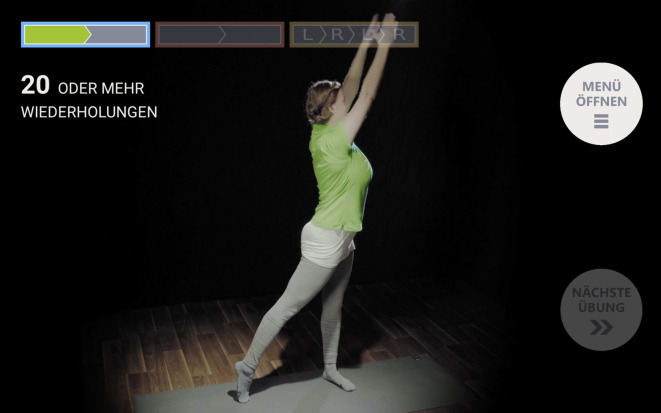

Die PmP nutzten die im Rahmen von ParkProTrain entwickelte videobasierte Tabletapplikation (App) im Anschluss an eine dreiwöchige stationäre PKB (Operationen- und Prozedurenschlüssel [OPS] 8‑97d) über einen Zeitraum von 9 Monaten in der Häuslichkeit. Ziel war es, dass die PmP die in der PKB erlernten körperlich aktivierenden Übungen regelmäßig weiterführen und die App sie bei der Etablierung eines eigenverantwortlichen und effektiven Trainings unterstützt. Das Trainingsprogramm enthält 122 Videos mit Parkinson-spezifischen Übungen aus den Bereichen Haltung, Kraft und Ausdauer. Der Therapeut erstellt mithilfe eines Administrationspanels individualisierte Trainingspläne aus dem Übungsportfolio. Hierbei werden jeweils drei Übungen zu einer Trainingseinheit zusammengestellt. Insgesamt werden drei Trainingseinheiten je Woche für einen bestimmten Zeitraum zu einem Trainingsplan zusammengestellt. Trainingstage werden nicht vorgegeben, jedoch kann ein Patient nur eine Trainingseinheit pro Tag starten. Der Therapeut empfängt über das Administrationspanel Daten zur Trainingshäufigkeit eines Patienten sowie dessen Bewertung einzelner Übungen. Diese Informationen kann der Therapeut für die Erstellung eines aktualisierten Trainingsplans nutzen, der an die jeweils aktuellen Bedürfnisse eines Patienten angepasst wird. Ein Patient kann ein Training über den Startbildschirm der App starten. Er wird zunächst nach seinem individuellen Wohlbefinden gefragt. Hierauf basierend wird entweder das vom Therapeuten hinterlegte Training oder aber ein alternatives Entspannungstraining angeboten. Anschließend wird dem Patienten eine Vorschau der entsprechenden Trainingseinheit angezeigt (Abb. 1). Der Patient hat die Wahl, dieses Training zu absolvieren oder ein alternatives Training zu wählen. Eine beispielhafte Trainingsansicht ist Abb. 2 zu entnehmen.

## Methodik

### Studiendesign

Im Rahmen des ParkProTrain-Projekts wurde eine Mixed-methods-Studie durchgeführt [17]. In einer quasirandomisierten prospektiven Längsschnittstudie erhielt die Interventionsgruppe (IG) das individualisierbare tabletbasierte Trainingsprogramm während und für 9 Monate nach der PKB, die Kontrollgruppe (KG) erhielt die Standard-PKB und die Standardversorgung danach. Die Evaluation der Intervention erfolgt durch eine schriftliche Befragung zu drei Messzeitpunkten (Beginn und Ende PKB sowie nach 9 Monaten). Darüber hinaus erfolgte eine qualitative Analyse von Interviews und Fokusgruppen hinsichtlich „feasibility“ und Akzeptanz. Eine derartige Kombination von Methoden ist in der empirischen Sozialforschung üblich. Ein Vorteil ist die komplementäre Ergänzung der Ergebnisse beider Methoden, um bspw. Erklärungslücken zu schließen [1]. Da die ParkProTrain-Intervention äußerst komplex ist, wurde dieser Ansatz im Rahmen der Studie gewählt. Der qualitative Teil bestand u. a. aus einer Befragung von PmP der Interventionsgruppe. Im vorliegenden Manuskript werden die Ergebnisse aus den qualitativen Patienteninterviews berichtet.

Die Rekrutierung der Studienteilnehmer erfolgte monozentrisch an einer Fachklinik für Parkinson und Bewegungsstörungen in Norddeutschland. Zunächst wurden die Patienten für die Kontrollgruppe und anschließend die Teilnehmer der Interventionsgruppe rekrutiert. Dieses sequenzielle Vorgehen wurde gewählt, da es aus organisatorischen Gründen nicht möglich war, beide Gruppen gleichzeitig in der Partnerklinik zu betreuen. Eingeschlossen wurden Patienten mit einem idiopathischen Parkinson-Syndrom (G20.0 und G20.1), die für eine dreiwöchige PKB in die Klinik aufgenommen wurden. Zudem erfolgte ein MoCA- (Montreal Cognitive Assessmemnt) und ein BBS-Screening (Berg Balance Scale). Patienten mit einem MoCa-Wert unter 18 Punkte und einem BBS-Wert unter 41 Punkten wurden von einer Studienteilnahme ausgeschlossen. Außerdem führten eine schwere depressive Episode, eine mittelgradige bis schwere Demenz sowie kardiovaskuläre und orthopädische/chirurgische sowie andere gesundheitliche Gründe zu Ausschlüssen. Darüber hinaus mussten die Patienten über ausreichende Deutschkenntnisse verfügen, um sowohl die Fragebögen ausfüllen als auch das Training mit der deutschsprachigen App durchführen zu können. Insgesamt wurden 127 Patienten in die Interventionsgruppe eingeschlossenen, von denen 93 Teilnehmer das neunmonatige Training komplett absolvierten. Die Patienten der Interventionsgruppe waren im Mittel 64,1 Jahre alt und die Krankheitsschwere nach Hoehn und Yahr betrug 2,57.

Alle IG-Teilnehmer nahmen im Rahmen ihrer PKB an drei Einführungsseminaren (zwei Einzelseminare und ein Gruppenseminar) teil und erhielten hier bereits ein mit der Trainings-App ausgestattetes Tablet, um den Umgang mit dem Programm zu erlernen. Vor der Entlassung hinterlegte die betreuende Physiotherapeutin einen individuellen Trainingsplan für die PmP. Die PmP wurden gebeten, nach Entlassung dreimal wöchentlich ein Training zu absolvieren. Außerdem sollte einmal wöchentlich eine frei wählbare Ausdaueraktivität durchgeführt und dokumentiert werden. Im Abstand von 3 Wochen fanden Gespräche mit der Physiotherapeutin statt (jeweils zwei Telefonate gefolgt von einem Vor-Ort-Termin in der Klinik^1^). Diese hatten zum Ziel, die PmP kontinuierlich zum Training zu motivieren. Zudem fand ein Austausch über die Machbarkeit des Trainings statt. Derartige Informationen über das Training halfen der Physiotherapeutin neben den übermittelten Trainingsdaten dabei, den Trainingsplan an die aktuellen Bedürfnisse der Studienteilnehmer anzupassen. Die Trainingspläne wurden alle 9 Wochen aktualisiert.

**Figure 3:**
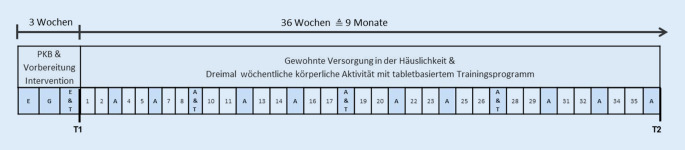

Die qualitativen, leitfadengestützten Interviews wurden mit 16 PmP zu zwei Zeitpunkten geführt. Das erste Interview erfolgte 9 Wochen nach PKB-Ende (T1), wenn die PmP bereits Erfahrungen mit dem ersten Trainingsplan gesammelt haben. Das zweite Interview erfolgte am Ende der Intervention 36 Wochen nach PKB-Ende (T2), um eine Einschätzung auf die gesamte Trainingszeit zu erhalten. In Abb. 3 ist der Ablauf der Studie für die Interventionsgruppe dargestellt.

Die PmP sollten bei den Kriterien Geschlecht, Krankheitsschwere, Bildungsniveau und körperliche Aktivität unterschiedliche Ausprägungen aufweisen (Tab. 1). Die Interviewpartner waren im Mittel 64,5 Jahre alt. Die Krankheitsschwere nach Hoehn und Yahr betrug durchschnittlich 2,69.

**Table 1:** 

Nr.	Geschlecht	Krankheitsschwere^a^	Bildungsniveau^b^	Körperliche Aktivität^c^	Patient	Status
1	Weiblich	Gering	Niedrig	Wenig	Frau 1	
2	Weiblich	Gering	Niedrig	Viel	n. A.	n. A.^d^
3	Weiblich	Gering	Hoch	Wenig	Frau 2	
4	Weiblich	Gering	Hoch	Viel	Frau 3	
5	Weiblich	Hoch	Niedrig	Wenig	Frau 4	
6	Weiblich	Hoch	Niedrig	Viel	Frau 5	
7	Weiblich	Hoch	Hoch	Wenig	Frau 6	
8	Weiblich	Hoch	Hoch	Viel	Frau 7	Drop-out^e^
9	Männlich	Gering	Niedrig	Wenig	Herr 1	
10	Männlich	Gering	Niedrig	Viel	Herr 2	
11	Männlich	Gering	Hoch	Wenig	Herr 3	
12	Männlich	Gering	Hoch	Viel	Herr 4	
13	Männlich	Hoch	Niedrig	Wenig	Herr 5	
14	Männlich	Hoch	Niedrig	Viel	Herr 6	
15	Männlich	Hoch	Hoch	Wenig	Herr 7	Drop-out^e^
15	Männlich	Hoch	Hoch	Wenig	Herr 8	
16	Männlich	Hoch	Hoch	Viel	Herr 9	

^a^Krankheitsschwere nach Hoehn-und-Yahr-Score: 0 bis 2,5 ≙ gering; > 2,5 ≙ hoch

^b^Bildungsniveau: Hauptschule oder kein Abschluss ≙ niedrig, höher als Hauptschule oder kein Abschluss ≙ hoch

^c^Körperliche Aktivität nach WHO-Empfehlung [13] mind. 2,5 h (150 min) pro Woche: < 150 min/Woche ≙ wenig, ≥ 150 min/Woche ≙ viel

^d^Es wurde kein passender Patient für diese Merkmalskombination gefunden

^e^Patient im Studienverlauf ausgeschieden (lediglich Interview zu T1 wurde geführt)

### Interviews

Die Interviews erfolgten anhand eines halbstandardisierten Interviewleitfadens. Themen waren 1. die Machbarkeit der Umsetzung des Trainings zu Hause und die Trainingsmotivation sowie der persönliche Nutzen und 2. die Zufriedenheit mit einzelnen Aspekten des Programms sowie weitere Wünsche und Bedürfnisse der PmP (Tab. 2).

**Table 2:** 

Themen der Interviewleitfäden		T1	T2
Machbarkeit, Motivation und persönlicher Nutzen	Zurechtkommen mit dem Programm und Etablierung des Trainings in den Alltag	•	•
	Veränderung der Trainingsroutine im Zeitverlauf		•
	Trainingsmotivation durch das Programm	•	•
	Veränderung der Motivation im Zeitverlauf		•
	Auswirkungen des regelmäßigen Trainings	•	•
	Beurteilung des Nutzens der Teilnahme	•	•
	Weiterempfehlung des Programms		•
Zufriedenheit und Wünsche	Zufriedenheit mit dem Trainingsplan	•	•
	Zufriedenheit mit der Gestaltung des Programms	•	•
	Zufriedenheit mit den Seminaren	•	
	Zufriedenheit mit den Telefonaten	•	•
	Zufriedenheit mit den Vor-Ort-Terminen	•	•
	Wünsche und Bedürfnisse	•	•
	Weiterführung des Trainings nach Studienende		•

*T1* 9 Wochen nach Parkinson-Komplexbehandlung, *T2* 36 Wochen nach Parkinson-Komplexbehandlung

Die Interviews wurden zwischen dem 10.10.2019 und 25.01.2021 vor Ort in der Klinik bzw. ab dem 12.03.2021 aufgrund der pandemiebedingten Kontaktbeschränkungen telefonisch durchgeführt.

### Datenanalyse

Die Interviews wurden digital aufgezeichnet und wörtlich transkribiert. Die Auswertung erfolgte mit dem Programm MAXQDA (12; 2020); es wurde die inhaltlich-strukturierende qualitative Inhaltsanalyse [11] angewandt. Inhaltlich relevante Themen wurden in Form eines Kategoriensystems systematisch beschrieben, die Hauptthemen (Oberkategorien) wurden deduktiv aus den Forschungsfragen und den Interviewleitfäden abgeleitet, Unterkategorien wurden induktiv durch Subsumption entwickelt [8].

## Ergebnisse

### Machbarkeit und Motivation

Alle PmP beschrieben das Training mit der App zu beiden Zeitpunkten als gut machbar. Sie bauten das Training in den ersten 9 Wochen auf verschiedene Art und Weise in ihren Alltag ein: Trainiert wurde zu bestimmten Tageszeiten oder an bestimmten Wochentagen. Insgesamt berichteten die PmP zum Interventionsende von einer sehr ähnlichen Trainingsroutine, die von drei PmP in ein eigenes Training integriert wurde.

Die Trainingsvideos der App halfen den PmP durch die visuelle und sprachliche Veranschaulichung zu beiden Zeitpunkten, die Übungen in der Häuslichkeit umzusetzen.

Ich habe so viel schon aufgeschrieben und gemacht an Bewegungsübungen. Ich habe jetzt 10 Jahre lang Parkinson. Aber das hat man aufgeschrieben, dann liegt das irgendwo im Ordner oder irgendwo drin. Und bei Ihnen da sieht man das und macht es einfach nach. Das ist also sehr gut, muss ich sagen. (Frau 4, Woche 9)

Zu Interventionsende konnten sechs PmP aufgrund ihrer gesundheitlichen Verfassung manche Übungen nicht immer wie vorgegeben durchführen. Hilfreich war in diesem Fall das auswählbare Alternativprogramm. Aufgrund der guten Passung des Trainings in den Alltag waren alle PmP dauerhaft motiviert, das Training durchzuführen. Vor allem zu T2 bemerkten alle Studienteilnehmer positive Wirkungen des regelmäßigen Trainings auf ihre körperliche Verfassung sowie jeweils zwölf auf die Bewältigung des Alltags und ihre wahrgenommene Lebensqualität. Der regelmäßige Kontakt und die Übermittlung der Trainingsdaten an die Physiotherapeutin lösten bei den Teilnehmern einen motivierenden, positiven Trainingsdruck aus. Die konkrete Vorgabe, dreimal wöchentlich nach einem definierten Trainingsplan zu trainieren, war für die PmP hilfreich. Ein wesentlicher Nutzen lag vor allem in der zeitlichen und örtlichen Flexibilität der Nutzung des Programms begründet. Die Tatsache, dass das Training zu Hause durchgeführt werden konnte, wurde vor allem während der Corona-Pandemie von drei PmP als positiv empfunden. Das Training war zeitweise die einzige durchführbare körperliche Aktivität.

Also auch generell bin ich sehr zufrieden mit dem Programm, eben auch weil man merkt, dass man zu Hause was machen kann. Ohne viel Geräte zu haben. Gerade jetzt in der Zeit ist das natürlich wichtig gewesen. Das auch mal zu merken, wie man zu Hause was macht, wenn man nicht in eine Praxis irgendwo gehen würde. (Frau 4, Woche 36)

Die Möglichkeit mit dem Partner zu trainieren, bewirkte ebenso wie ein neuer Fokus der Übungen und verschiedene Details im Programm eine Trainingsmotivation. Technische Probleme, von denen von neun PmP zu T1 berichtet wurde, führten zwar nicht zur Demotivation, wurden jedoch als unerfreulich beschrieben.

Alle bis auf einen Studienteilnehmer würden das Programm anderen PmP weiterempfehlen. Neben den genannten motivierenden Eigenschaften wurde von einem Patienten ergänzend erwähnt, dass die wahrgenommene Fundiertheit des Programms Grund für eine Empfehlung ist.

Macht da mit! Das würde ich durchaus ohne Zweifel sagen. Macht da mit, da habt ihr ein Video mal an die Hand bekommen, das gut fundiert ist. Die Leute, die da vorturnen und die die Übungen sich ausgedacht haben und sie ausführen, die haben da Ahnung von. Die wissen was gut ist und das ist gut für euch. Also das würde ich Parkinsonleuten weiterempfehlen. (Herr 3, Woche 36)

### Zufriedenheit mit den Einführungsseminaren in der Klinik

Mit den Seminaren während der PKB waren alle PmP zufrieden. Sie fühlten sich gut auf die Interventionszeit in der Häuslichkeit vorbereitet. Die Seminare haben den PmP vor allem geholfen, die Technik zu verstehen und dienten der Besprechung des ersten Trainingsplans. Außerdem wurde die Bedeutung von körperlicher Aktivität und Bewegung bei Parkinson adressiert.

### Zufriedenheit mit den Telefonaten und den Vor-Ort-Terminen

Der regelmäßige Austausch mit der Physiotherapeutin wurde zu T1 von allen bis auf zwei und zu T2 von allen bis auf einen Befragten als hilfreich beschrieben. Die Häufigkeit der Telefongespräche alle 3 Wochen beurteilten die meisten Befragten zu T2 rückblickend als angemessen. Ein Patient hätte sich zu T2 rückblickend noch mehr Kontakt gewünscht. Dies begründet er jedoch mit dem angenehmen persönlichen Kontakt und nicht mit einem Mehrbedarf an Unterstützung beim Training. In erster Linie hatten die Telefonate eine motivierende Wirkung auf die PmP. Sie boten Raum, über einzelne Übungen zu sprechen. Zu T1 fokussierten die Rückfragen auf die Umsetzung des Trainings, zu T2 stand die Besprechung der erfolgversprechendsten Übungen im Vordergrund.

Da war jemand, der sich erkundigte, wie es mir geht. Ob ich mit dem Sport klarkomme oder nicht. Ob es da Verbesserung oder was anderes gibt. Ich fühlte mich aufgehoben, ich hatte jemanden. Das ist der Motivator. (Frau 3, Woche 36)

Die Vor-Ort-Termine in der Klinik boten Gelegenheit, ausführlich über das Training der vergangenen Wochen zu sprechen. Dies half bei der gemeinsamen Gestaltung des jeweils neuen Trainingsplans. Die Vor-Ort-Termine wurden als persönlicher wahrgenommen als die Telefonate. Die Selbstreflektion war zudem vorab intensiver. Allerdings erwähnten vier PmP, dass die Termine in der Klinik bei weiter Anreise schwierig umzusetzen sind. Die vier PmP, die aufgrund der Pandemie keine Vor-Ort-Termine wahrnehmen konnten, sind mit den Telefonaten gut zurechtgekommen.

### Zufriedenheit mit dem Trainingsplan und den Übungen

Das Trainingsprogramm wurde zu T1 als abwechslungsreich beschrieben. Für einige PmP war es hilfreich, wenn ihnen Übungen teilweise schon bekannt waren, andere profitierten vor allem von neuen Übungen. Der Schweregrad der Übungen wurde zu Beginn der Intervention unterschiedlich wahrgenommen. In fünf Fällen waren es einzelne Übungen oder spezifische Haltungen, die Schwierigkeiten bereiteten. Die PmP profitierten zu beiden Zeitpunkten davon, dass die Übungen speziell für PmP und an die aktuelle Tagesverfassung angepasst zusammengestellt wurden. Damit waren die Befragten über die gesamte Interventionszeit hinweg überwiegend zufrieden. Zwei PmP hätten sich zum Ende der Intervention mehr Abwechslung gewünscht. Spaß am Trainieren mit ihrem individualisierten Plan verspürten die PmP zu beiden Zeitpunkten.

### Zufriedenheit mit der App

Insgesamt kamen die PmP mit der Technik gut zurecht. Die App wurde von den Befragten zu T1 mit Begriffen wie „benutzerfreundlich“ und „kann jeder bedienen“ umschrieben. Die Darstellung der Videos wurde als professionell bewertet, die Videos mit der Physiotherapeutin als anspornend. Die PmP lobten zu T2 die Darstellung der Physiotherapeutin in den Videos von allen Seiten, da man so die korrekte Bewegung erkennt. Die sprachlichen Übungsanweisungen mit Wiederholungsmöglichkeit wurden als hilfreich bewertet.

In den ersten 9 Wochen der Intervention traten bei drei PmP lediglich kleinere Bedienungsschwierigkeiten auf, die mit der Physiotherapeutin geklärt werden konnten. Bei neun PmP traten zudem technische Fehler auf (z. B. Tonausfall), trotzdem war ein Training möglich. Von technischen Fehlern wurde am Ende der Intervention nur noch von vier PmP berichtet.

### Wünsche und Bedürfnisse

Sieben PmP äußerten zu T1 den Wunsch, häufiger als dreimal wöchentlich trainieren zu können. Dieser Wunsch wurde zu T2 lediglich noch von zwei aktiven PmP geäußert, die sich durch die dreimal wöchentliche Trainingsvorgabe ausgebremst fühlten. Die PmP waren sich zu T1 uneinig bzgl. der Pausenlänge zwischen den einzelnen Übungen. Während diese für einen PmP genau die richtige Länge hatten, empfanden zwei sie als zu lang. Darüber hinaus wurde ein Wunsch nach weiterer Individualisierbarkeit der eingesetzten Kleingeräte, einer Ansage der Atmung sowie der genauen Ausführung der Bewegungen in den Videos geäußert. Es fehlte die Möglichkeit, eine in der Vorwoche absolvierte Ausdauertätigkeit nachträglich sowie mehr als eine Einheit wöchentlich einzutragen. Eine Einsicht in bereits absolvierte Einheiten hätten Gespräche mit der Physiotherapeutin über absolvierte Übungen erleichtert. Es wurden weitere Verbesserungsideen geäußert: eine genauere Differenzierung der Bewertung einer Übung, eine weniger umfangreiche Einschätzung der eigenen Tagesverfassung, eine größere Ansicht der Trainingsvorschau sowie eine Behebung der technischen Fehler.

Aus den von den Interviewpartnern genannten positiven und negativen Aspekten des Trainingsprogramms können Erfolgsfaktoren und Barrieren abgeleitet werden (Tab. 3).

**Table 3:** 

	Erfolgsfaktoren	Barrieren
Machbarkeit	– Örtlich und zeitlich flexibles Training – Trainingsanpassung an jede Tagesverfassung – Möglichkeit des Trainings mit Partner – Motivation durch positive Trainingseffekte – Motivation durch Festlegung auf dreimal wöchentliches Training	– Ausbremsung aktiver Patienten durch Festlegung auf dreimal wöchentliches Training
Trainingsplan und Übungen	– Hilfreiche und anschauliche Trainingsvideos – Einbezug weniger Trainingsutensilien – Wahrgenommene Fundiertheit der Übungen – Parkinson-Bezug der Übungen – Abwechslung durch neue Übungen – Vertrautheit durch bekannte Übungen – Pausenlänge zwischen Übungen für einige genau richtig	– Übungen nicht fordernd genug für aktivere Patienten – Zu wenig Einbezug von in der Häuslichkeit verfügbaren Kleingeräten – Schwierigkeiten mit bestimmten Haltungen – Pausen zwischen Übungen für einige zu lang – *Fehlende Ansagen hinsichtlich:* – Der Atmung bei der Übungsausführung – Der konkreten Bewegungsausführung
Therapeutische Betreuung	– *Einführungsseminare vermittelten:* – Bedeutsamkeit körperlicher Aktivität – Umgang mit dem System – Gefühl von guter Vorbereitung auf Intervention – Regelmäßige Telefonate waren motivierend und hilfreich – *Regelmäßige Vor-Ort-Termine:* – Förderten intensive Selbstreflektion der Patienten – Wurden als persönlicher als Telefonate empfunden – veranschaulichten Übungen und Trainingspläne – Motivation durch Trainingsdatenübermittlung	– Eingeschränkte Machbarkeit der Vor-Ort-Termine bei weiter Anreise
Technische Umsetzung	– Einfache Erlernbarkeit und Handhabbarkeit des Programms – Möglichkeit der Wahl eines Alternativtrainings	– Mangelnde Einsicht in absolvierte Trainings – Ausdauereinheit nicht nachträglich für vergangene Woche und lediglich einmal eintragbar – Bewertung von Übungen nicht differenziert genug – Einschätzung der Tagesverfassung zu differenziert – Technikfehler – Anfangs: fehlende Regulierung der Lautstärke der Ansagen

## Diskussion

Im Folgenden werden aus den identifizierten Erfolgsfaktoren und Barrieren vier zentrale Empfehlungen für eine zukünftige Umsetzung ähnlicher Vorhaben abgeleitet. Außerdem werden Stärken und Limitationen der Untersuchung aufgeführt.

### Empfehlung 1: Parkinson-Spezifität und Individualisierbarkeit

Physiotherapie bei Parkinson sollte fortlaufend an die individuellen Bedürfnisse der PmP angepasst werden [5]. In ParkProTrain wird diese Empfehlung der S3-Leitlinie durch die Erstellung individualisierter Trainingspläne alle 9 Wochen umgesetzt. Zudem wird die Wahl eines Entspannungs- oder Alternativtrainings je nach aktueller Tagesverfassung ermöglicht. Eine derartige Anpassbarkeit kann bedeutsam sein, wenn PmP im Tagesverlauf einen häufigen Wechsel der Leistungsfähigkeit durchlaufen [9, 19].

Zusätzlich zu den Individualisierungsmöglichkeiten, die das Programm bietet, werden weitere Wünsche geäußert. So wäre eine Individualisierbarkeit der Pausenlänge zwischen einzelnen Übungen denkbar. Zudem scheint eine Ausrichtung der Anzahl der wöchentlichen Trainings- und Ausdauereinheiten an dem persönlichen Aktivitätslevel für zukünftige Vorhaben erstrebenswert. Das individuell besprochene Trainingsziel sollte dabei weiterhin machbar für den jeweiligen PmP sein [10].

Die Übungen wurden von den PmP hinsichtlich Intensität und Aufbau unterschiedlich wahrgenommen und bewertet. Das Programm bietet mit seinen 122 unterschiedlichen Übungseinheiten [16] und der Möglichkeit des Einbezugs von Thera-Bändern, Stühlen, Nordic-Walking-Stöcken und Wasserflaschen oder vorhandener Kleingeräte bereits vielfältige Möglichkeiten, auf individuelle Wünsche einzugehen. Dennoch hat dieses große Übungsportfolio Erweiterungspotenzial.

Die wahrgenommene Fundiertheit der Übungen wirkte sich positiv auf die Trainingsmotivation aus. Diese sowie die Individualisierbarkeit werden auch in einer anderen Studie als wichtige Aspekte für eine erfolgreiche Implementierung regelmäßiger körperlicher Aktivität bei PmP berichtet [18].

Die Möglichkeit, den Partner in das Training einzubeziehen, stellt einen weiteren bedeutsamen Vorteil des Programms dar. In der S3-Leitlinie IPS wird eine Einbeziehung des Partners in die physiotherapeutische Behandlung ausdrücklich empfohlen [5]. Studien bei anderen Indikationen belegen, dass sich ein Einbezug des sozialen Netzwerks in die Nachsorge positiv auf die sportliche Aktivität auswirken kann [15].

### Empfehlung 2: Örtlich und zeitlich flexibles Training

Das örtlich und zeitlich unabhängige Training ist ein Vorteil, den das Trainingsprogramm bspw. gegenüber einer ambulanten Therapie in einer Praxis aufweist. Ein örtlich flexibles Training kann durch den Einsatz portabler Endgeräte gewährleistet werden. Hierdurch kann eine Trainingsunterbrechung bei einer Reise oder einem Klinikaufenthalt der PmP verhindert werden. Ein Training von zu Hause aus machte während der Corona-Pandemie körperliche Aktivität unabhängig von Öffnungen im Fitness- und Therapiebereich möglich, was sich als weiterer Vorteil dieses kontaktlosen, videobasierten Programms herausstellte. Vor allem, da erste Quellen eine eingeschränkte körperliche Aktivität während der Pandemie belegen [12].

### Empfehlung 3: Enge und persönliche Betreuung

Eine geringe Erwartung an Erfolge körperlicher Aktivität kann sich bei PmP aktivitätshemmend auswirken [7]. Es wird empfohlen, PmP zu Beginn einer physiotherapeutischen Intervention extrinsisch zum Training zu motivieren [10]. Die Bedeutsamkeit regelmäßig angepasster körperlicher Aktivität bei Parkinson wurde bereits vor Interventionsstart im Rahmen der Einführungsseminare in der Klinik vermittelt. Auch der regelmäßige Kontakt und die Übermittlung der Trainingsdaten hatten motivierende Wirkung auf die PmP. Die teilweise weite Anreise zu den Vor-Ort-Terminen wurde nicht nur von den Interviewpartnern als herausfordernd erwähnt, sondern war auch der meist genannte Verweigerungsgrund während der Rekrutierung der Studienteilnehmer. Es stellten sich aber bedeutsame Vorteile der persönlichen Treffen gegenüber den Telefonaten heraus. Ein einmaliger Termin zur Hälfte der Interventionszeit für zukünftige Vorhaben könnte einen geeigneten Kompromiss darstellen. Eine andere Möglichkeit der Umsetzung persönlicherer Treffen wäre die Durchführung mehrerer videobasierter Kontakte über die Interventionszeit hinweg.

### Empfehlung 4: Einfache Erlernbarkeit und Handhabbarkeit der Technik

Eine einfache Handhabbarkeit ist Bestandteil bekannter Technikakzeptanzmodelle [4]. Um das System entsprechend nutzerfreundlich zu gestalten, wurden die PmP im Rahmen eines User-centered-design-Ansatzes in die Entwicklung eingebunden [16]. Die einführenden Seminare sowie die Nutzungsmöglichkeit während der PKB waren darüber hinaus bedeutsam für die Vermittlung der Funktionalitäten der App. Die als anschaulich und hilfreich wahrgenommenen Trainingsvideos führten zu einer unkomplizierten Umsetzung des Trainings zu Hause. Ergänzt werden könnte das Programm um die von den PmP genannten Aspekte, wie bspw. eine noch detailliertere Ansage einzelner Bewegungsausführungen oder die Einsicht in bereits absolvierte Trainings.

### Stärken und Limitationen

Die Teilnehmer der Interventionsgruppe nutzten die Trainings-App während der Corona-Pandemie. Die Äußerungen der Interviewpartner deuten darauf hin, dass das Training mit dem Programm teilweise die einzige körperliche Aktivität darstellte, der die PmP nachgingen. Hierin können, wie in Empfehlung 2 formuliert, Vorteile begründet liegen. Es wäre aber auch denkbar, dass die Ergebnisse der Interviews nicht ausschließlich auf eine pandemiefreie Zeit übertragbar sind. Patienten der Interventionsgruppe hatten ggf. durch einen Mangel an anderen Möglichkeiten mehr Zeit, sich dem Trainingsprogramm zu widmen. Dies könnte zu einer insgesamt positiveren Auffassung des Programms geführt haben. Negative Gestimmtheit während der Pandemie könnte ggf. aber auch zu einer insgesamt negativeren Bewertung des Programms geführt haben.

## Fazit für die Praxis

- Ein tabletbasiertes Trainingsprogramm ist geeignet, um ein kontinuierliches Eigentraining in der Häuslichkeit bei PmP zu unterstützen.
- Umfassende Einführungsseminare sind wichtige Grundlage für eine erfolgreiche Umsetzung.
- Das richtige Verhältnis aus festen Vorgaben und örtlich und zeitlich flexiblem Training ist bedeutsam.
- Eine regelmäßige Betreuung durch einen vertrauten Therapeuten ist ratsam.
- Eine Veranschaulichung von Übungen durch detailreiche Videos ist wesentlich.
- Eine Ausrichtung auf Parkinson-spezifische und individuelle Bedürfnisse sollte fokussiert werden.
- Ein Einbezug von Angehörigen in das Training kann motivierend wirken.

## Footnotes

1 Aufgrund der Kontaktbeschränkungen während der Corona-Pandemie fanden alle Gespräche ab Mitte März 2020 telefonisch statt.
