# Erratum zu: Implementierung eines individualisierbaren tabletbasierten Trainingsprogramms im Anschluss an eine Parkinson-Komplexbehandlung in der Häuslichkeit – Erfolgsfaktoren und Barrieren

**DOI:** 10.1007/s00115-022-01368-1

**Published:** 2022-08-08

**Authors:** Lynn Wagner, Ruth Deck

**Affiliations:** Institut für Sozialmedizin und Epidemiologie, Universität zu Lübeck, Ratzeburger Allee 160, 23562 Lübeck, Deutschland

**Erratum zu:**

**Nervenarzt 2021**

10.1007/s00115-021-01202-0

Im Artikel „Implementierung eines individualisierbaren tabletbasierten Trainingsprogramms im Anschluss an eine Parkinson-Komplexbehandlung in der Häuslichkeit – Erfolgsfaktoren und Barrieren“ von L. Wagner und R. Deck fehlen bei den Abbildungen 1 und 2 die Quellenhinweise. Für beide Abbildungen lautet der Quellenverweis: Schlussbericht ParkProTrain, Förderkennzeichen 01VSF17037, Abb. 9 und 10, S. 12.

Weiter möchten die Autorinnen über die Quellenangabe [16] hinaus konkretisieren, dass die verwendete App vom Institut für Telematik (ITM) und vom Institut für Multimediale und Interaktive Systeme (IMIS) der Universität zu Lübeck erstellt wurde. Die Fachklinik für Parkinson und Bewegungsstörungen der Segeberger Kliniken war für die Konzeption der Übungsinhalte der Videos sowie die Rekrutierung und Betreuung der Patient*innen verantwortlich.

Eine fehlende Danksagung wird mit diesem Acknowledgement ergänzt:

Die Autorinnen danken dem Institut für Telematik (Prof. Dr. Andreas Schrader), dem Institut für Multimediale und Interaktive Systeme der Universität zu Lübeck (Prof. Dr. Nicole Jochems) für die Entwicklung und Gestaltung der App sowie der Fachklinik für Parkinson und Bewegungsstörungen, Segeberger Kliniken (Prof. Dr. Björn Hauptmann; Frau Ann-Kristin Hoffmann), für die Rekrutierung und die Betreuung der teilnehmenden Patient*innen.

